# Supplementary material for: Determinants of gastric cancer immune escape identified from non-coding immune-landscape quantitative trait loci
Source: Nat Commun. 2024 May 21;15:4319. doi: 10.1038/s41467-024-48436-5 (PMC11109163; doi:10.1038/s41467-024-48436-5)
Supplement: Supplementary file 1 — Supplementary Information [file 41467_2024_48436_MOESM1_ESM.pdf]

Supplementary Information

Determinants of gastric cancer immune escape identified from non-coding immune-landscape quantitative trait loci

Christos Miliotis[1,2], Yuling Ma[1,3], Xanthi-Lida Katopodi[1], Dimitra Karagkouni[1,3,4], Eleni Kanata[1], Kaia Mattioli[1,5], Nikolas Kalavros[1,3,6], Yered H. Pita-Juárez[1,3], Felipe Batalini[1,7], Varune R. Ramnarine[1], Shivani Nanda[1,3,4], Frank J. Slack[1,4,*], and Ioannis S. Vlachos[1,3,4,6,*]

(1) Harvard Medical School Initiative for RNA Medicine, Department of Pathology, Beth Israel Deaconess Medical Center, Harvard Medical School, Boston, MA, USA.

(2) Harvard Program in Virology, Harvard University Graduate School of Arts and Sciences, Boston, MA, USA.

(3) Broad Institute of MIT and Harvard, Cambridge, MA, USA

(4) Cancer Center / Cancer Research Institute, Beth Israel Deaconess Medical Center, Harvard Medical School, Boston, MA, USA.

(5) Current address: Division of Genetics, Department of Medicine, Brigham and Women's Hospital and Harvard Medical School, Boston, MA, USA

(6) Spatial Technologies Unit, Beth Israel Deaconess Medical Center, Boston, MA, USA

(7) Current address: Division of Oncology, Department of Medicine, Mayo Clinic, Phoenix, AZ, USA

*Correspondence to: Ioannis S. Vlachos and Frank J. Slack

Email: (ivlachos@bidmc.harvard.edu , fslack@bidmc.harvard.edu)

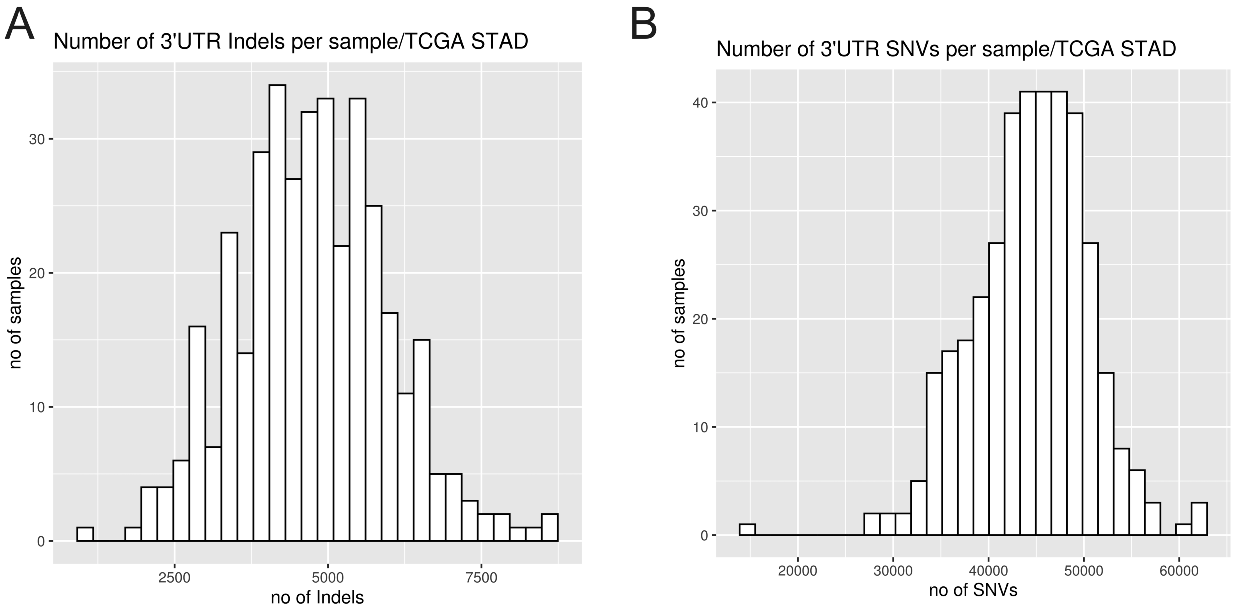

Supplementary Figure 1. Per-sample number of 3'UTR variants. (A-B) Distribution of the number of 3'UTR (A) short indels and (B) SNVs per sample called by the GATK variant calling analysis of TCGA STAD RNAseq data. Figure data are provided in the Source Data file.

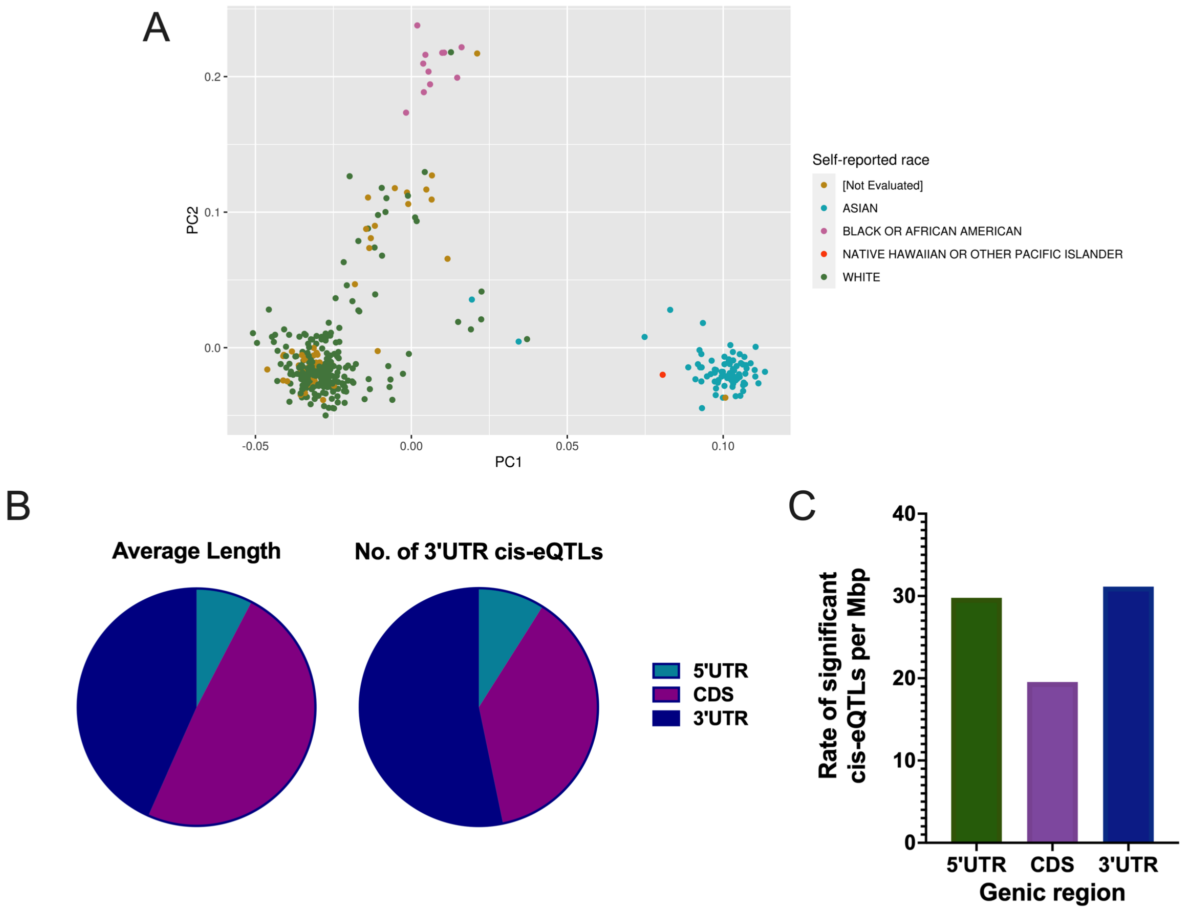

Supplementary Figure 2. Enrichment of significant *cis*-eQTL variants in 3'UTRs. (A) Plot of the first two genetic PCs based on WES-derived germline variants from TCGA STAD samples. Each sample is colored by self-reported race. (B) Comparing the average length of each genic region (5'UTR, CDS, 3'UTR) to the number of significant cis-eQTLs residing in those regions, reveals an enrichment of significant variants in the 3'UTR (chi-square p-value < 1e-5). (C) Rate per Mbp of significant cis-eQTLs in each genic region (5'UTR, CDS, 3'UTR). The rate was calculated by dividing the absolute number of significant cis-eQTLs mapping to each genic region divided by its collective genomic length. Figure data are provided in the Source Data file.

A

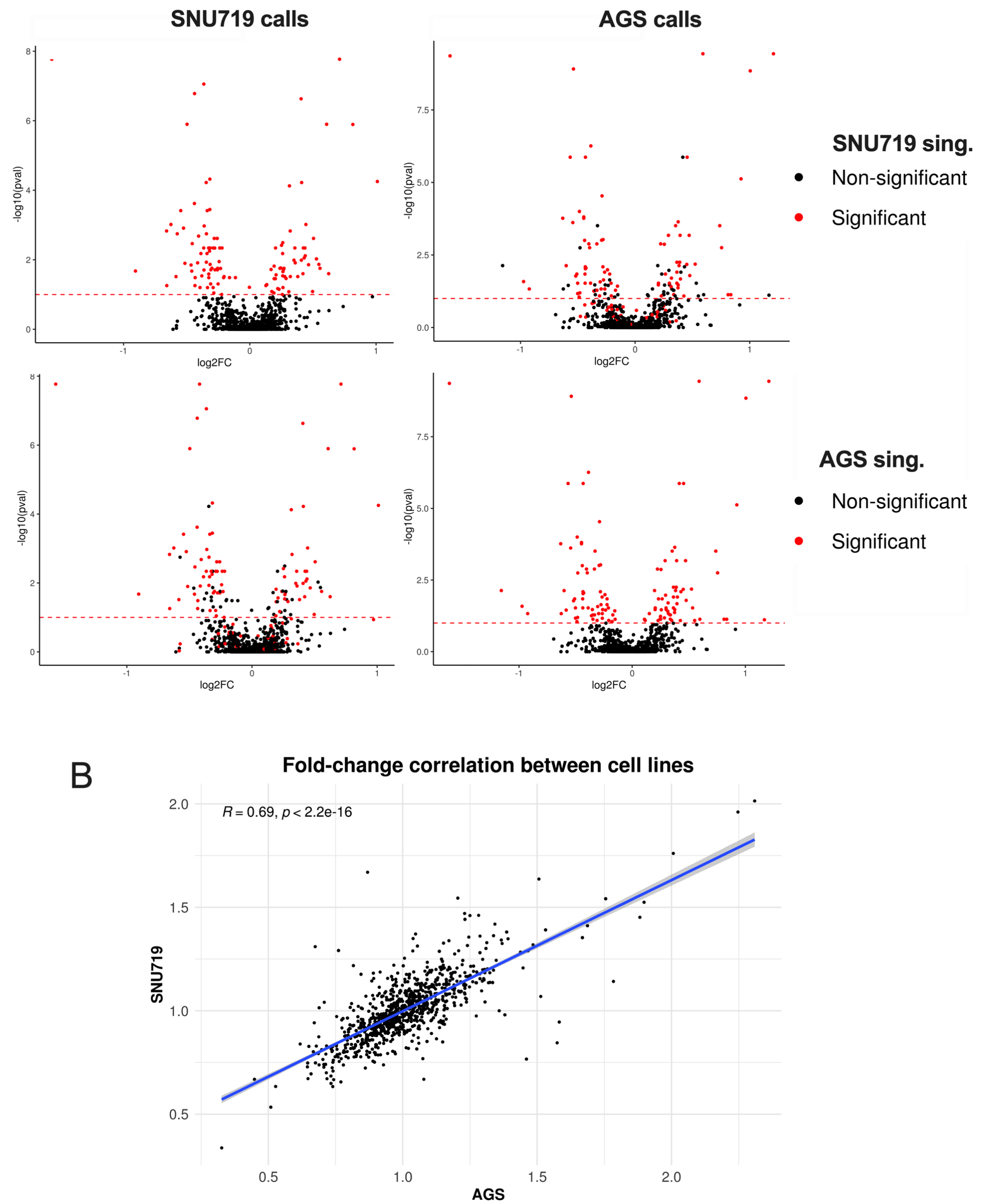

Supplementary Figure 3. Comparison of the MPRA results from AGS and SNU719 cell lines. (A) Volcano plots for the results from the MPRA assay with SNU719 (first column) and AGS (second column) cells. The dotted red line defines the cutoff for a significant call, any dots above the line have an adjusted p-value lower than 5e-2. Significant calls from the SNU719 assay are colored red in the first row, while significant calls from the AGS assay are shown in red in the second row. (B) Correlation between MPRA effect sizes in SNU719 and AGS cell lines (Pearson correlation coefficient $R = 0.69$, $p < 2.2e-16$, N=860 variants per cell line). Figure data are provided in the Source Data file.

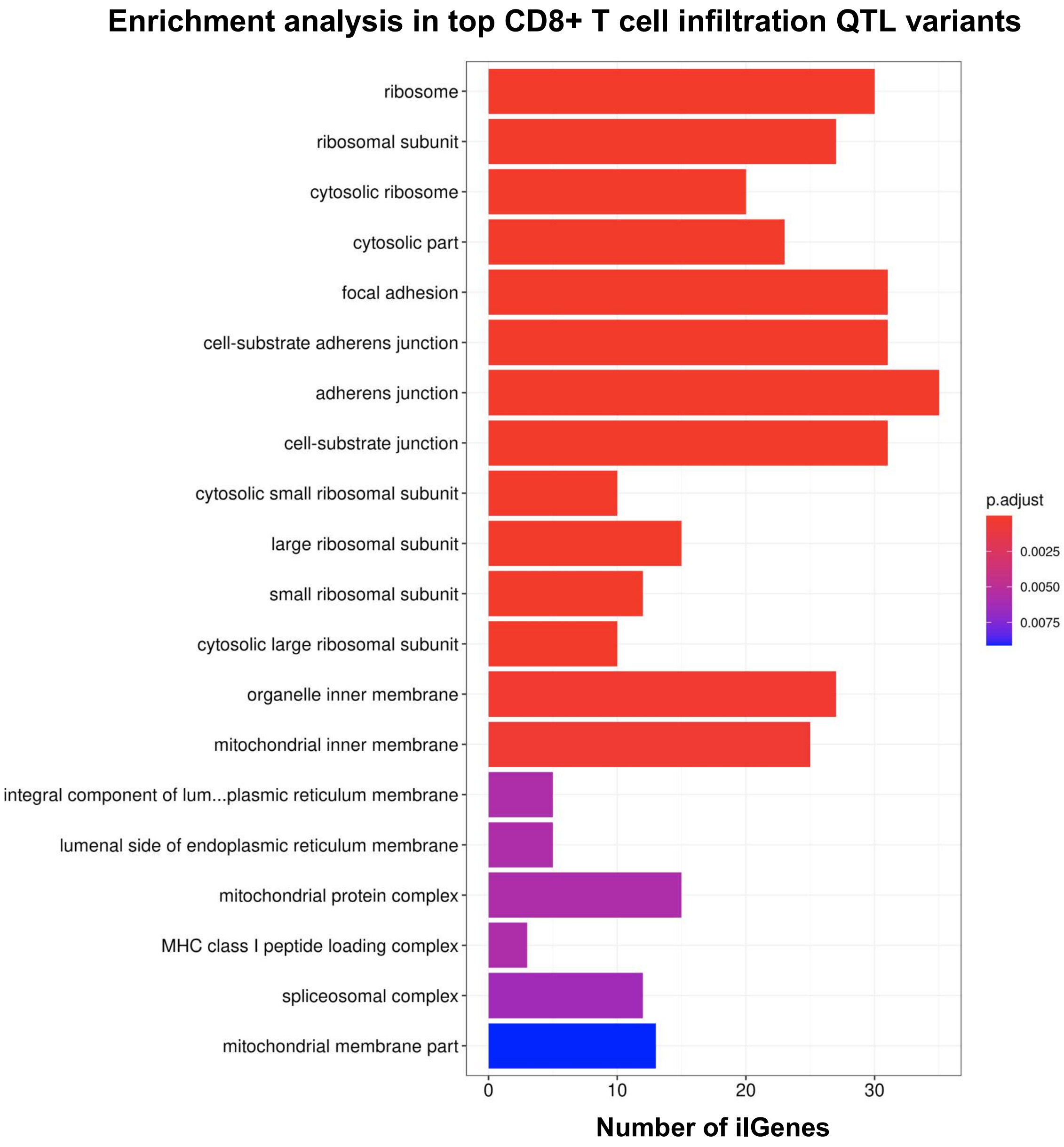

Supplementary Figure 4. Enrichment analysis in top CD8+ T cell infiltration QTL variants. Gene ontology (GO) cellular components (CC) analysis in genes with significant variants (N=467) as CD8+ T cell infiltration QTLs reveals enrichment in ribosome-related pathways. One-sided Fisher's exact test was utilized to evaluate cell type-specific enrichment of ilQTL genes. Figure data are provided in the Source Data file.

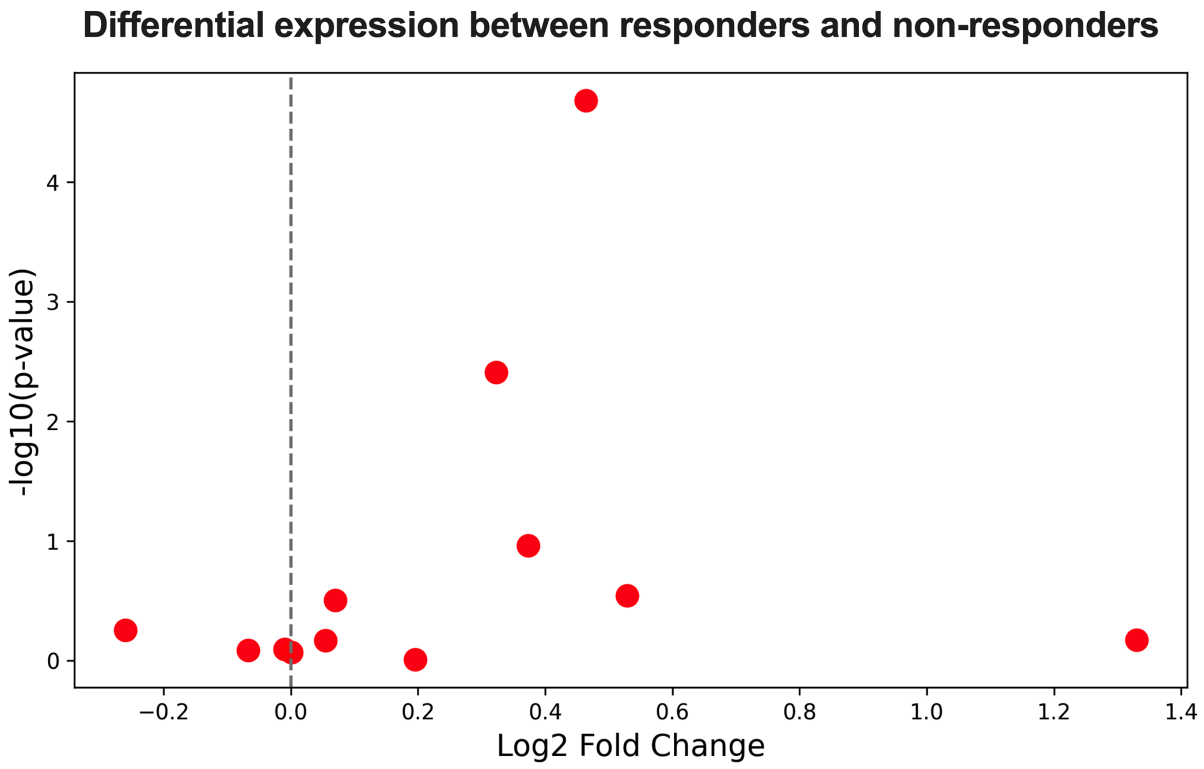

Supplementary Figure 5. ADAR overexpression is common in responders to immune-checkpoint inhibition. Differential gene expression log2(fold change) (x axis) and -log 10 p-value for baseline ADAR1 expression between responders and non-responders across 12 immune checkpoint inhibition studies. Log2(fold change) = 0, signifying equal expression between the two groups, has been marked with a vertical dashed line. Figure data and sample sizes are provided in the Source Data file.

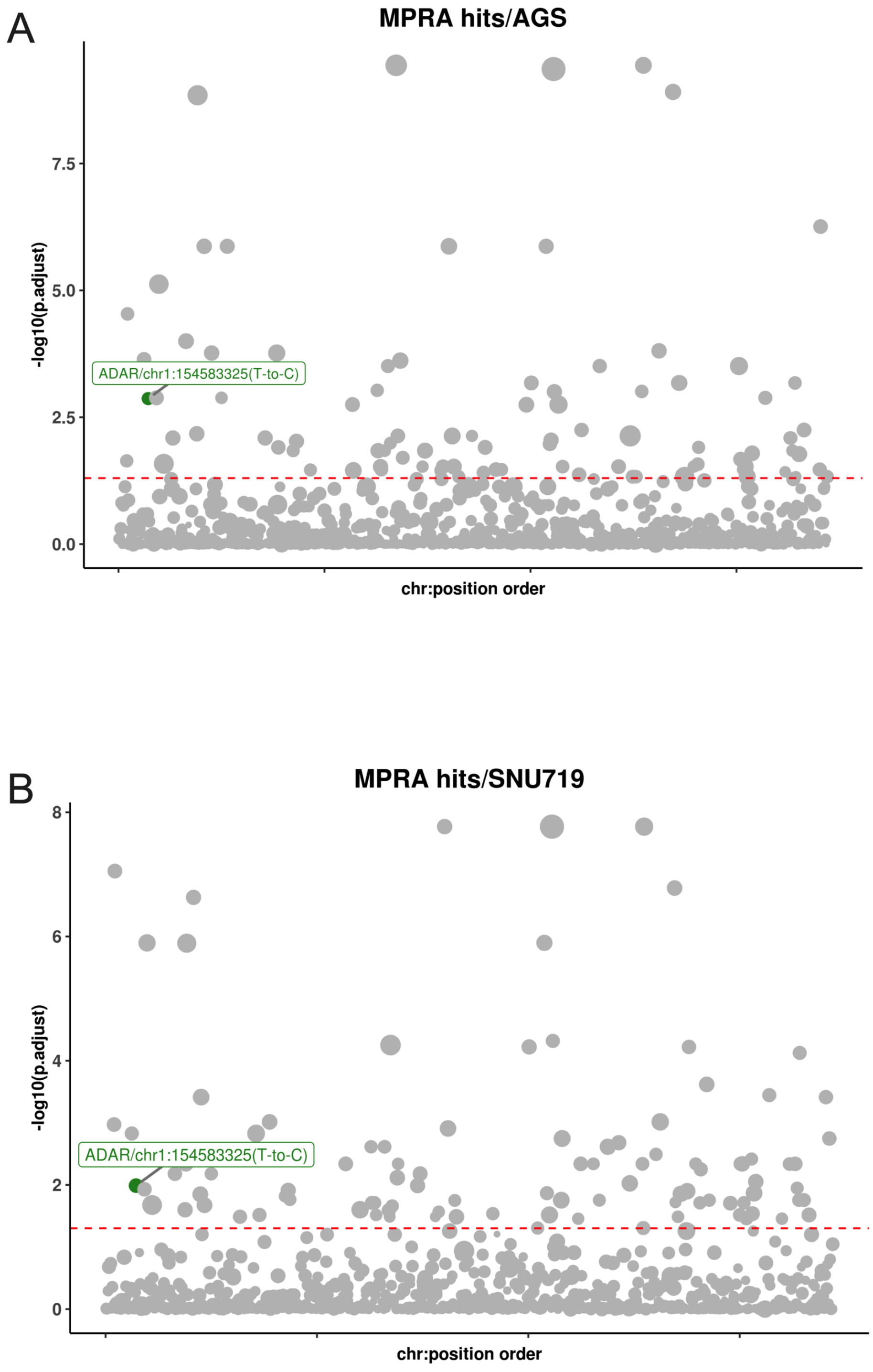

Supplementary Figure 6. ADAR eQTL and ilQTL variant appears as hit in MPRA assay. Manhattan plots of the results of the 3′UTR MPRA assay in the two gastric cancer cell lines (N=860 variants per cell line), (A) AGS and (B) SNU719. The point size for each variant increases with increasing absolute log2FC. The ADAR chr1:154583325 (T-to-C) variant is colored green, while all other variants are colored grey. Dots above the dashed red line (p.adjust=log2(0.05)) represent significant variants. Figure data are provided in the Source Data file.

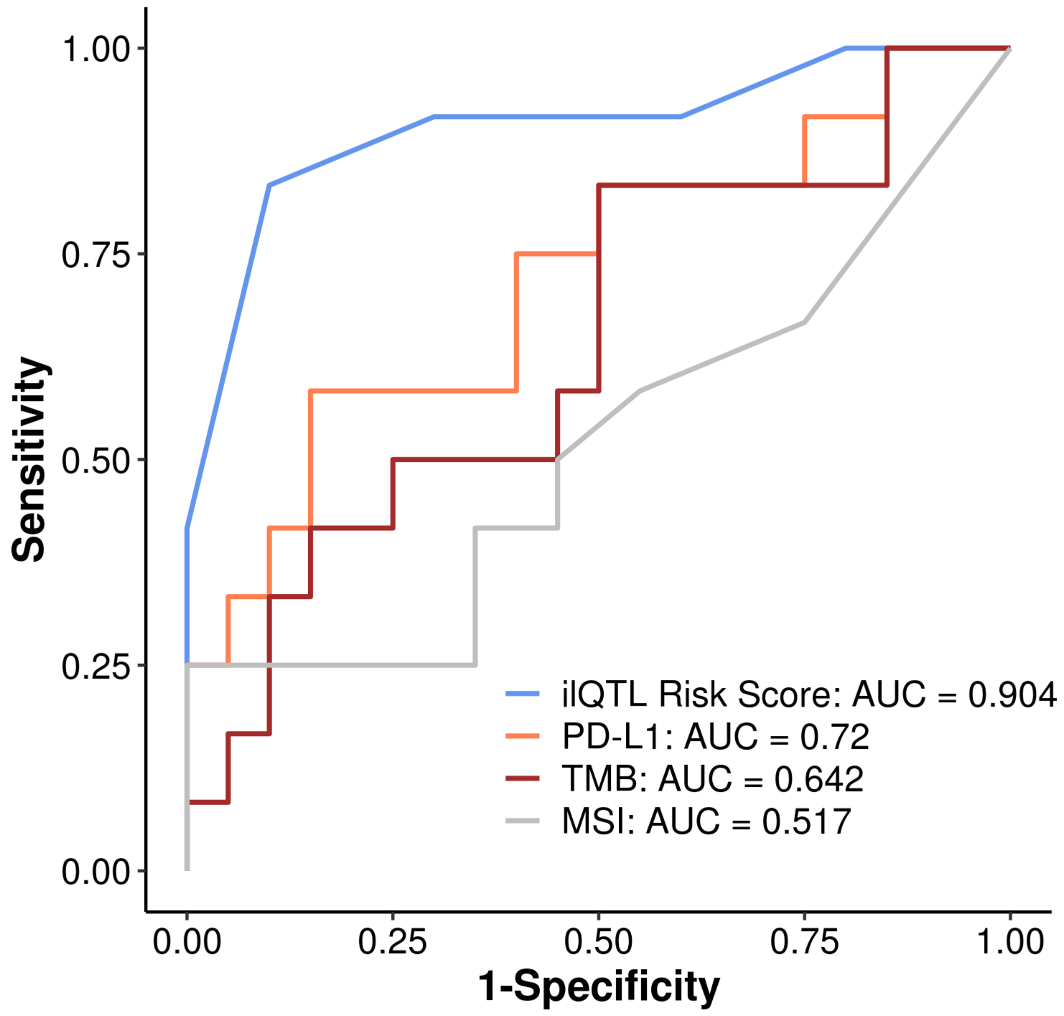

Supplementary Figure 7. Comparison of ilQTL 3' UTR PRS with PD-L1 expression classification, mutational burden and MSI in distinguishing responders and non-responders to ICI. Receiver Operator Characteristic (ROC) curves showing the ability of the TMB, MSI and PD-L1 expression classification to distinguish between R and NR patients in a subset of 32 patients from the PRS test set, where WES data were available. The Area Under the Curve (AUC) score is reported for all predictors and models. Figure data are provided in the Source Data file.

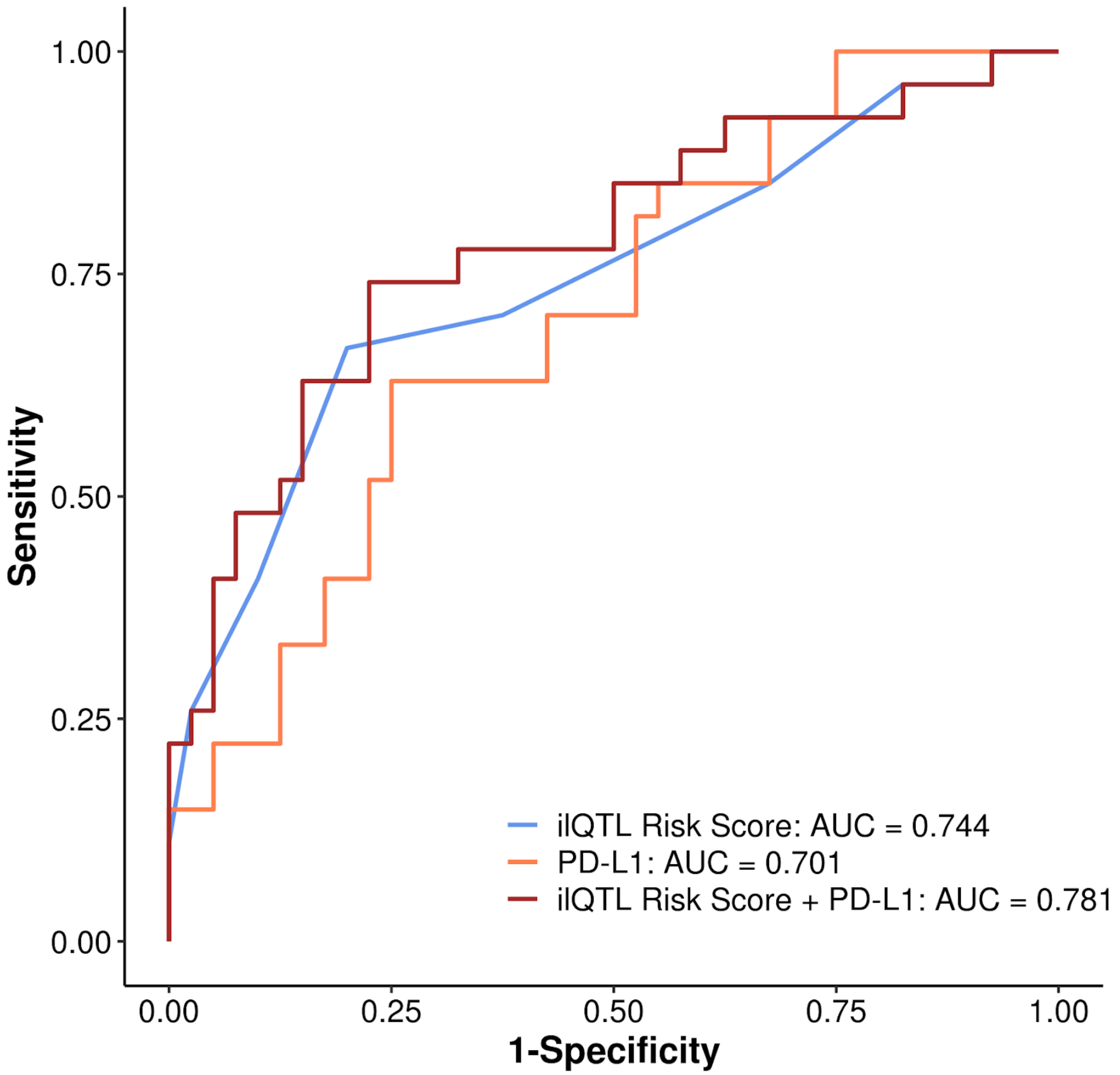

Supplementary Figure 8. Comparison of an ilQTL 3' UTR PRS with PD-L1 expression classification and a multivariate ilQTL + PD-L1 model in distinguishing responders and non-responders to ICI. Receiver Operator Characteristic (ROC) curves showing the ability of the PRS score, PD-L1 expression classification, and their linear combination with a regression model to distinguish between R and NR patients in the testing population (n=67). An Area Under the Curve (AUC) score is reported for all classifiers. The model combining the ilQTL PRS and PD-L1 expression presents the highest area under the receiver operating characteristic curve (AUC = 0.782), while both PD-L1 expression and ilQTLs were identified as independent significant predictors (PD-L1 expression: coef. 0.02, p=0.0136, ilQTLs PRS: coef. 0.05, p=0.0038, Multivariate Linear Regression). Figure data are provided in the Source Data file.

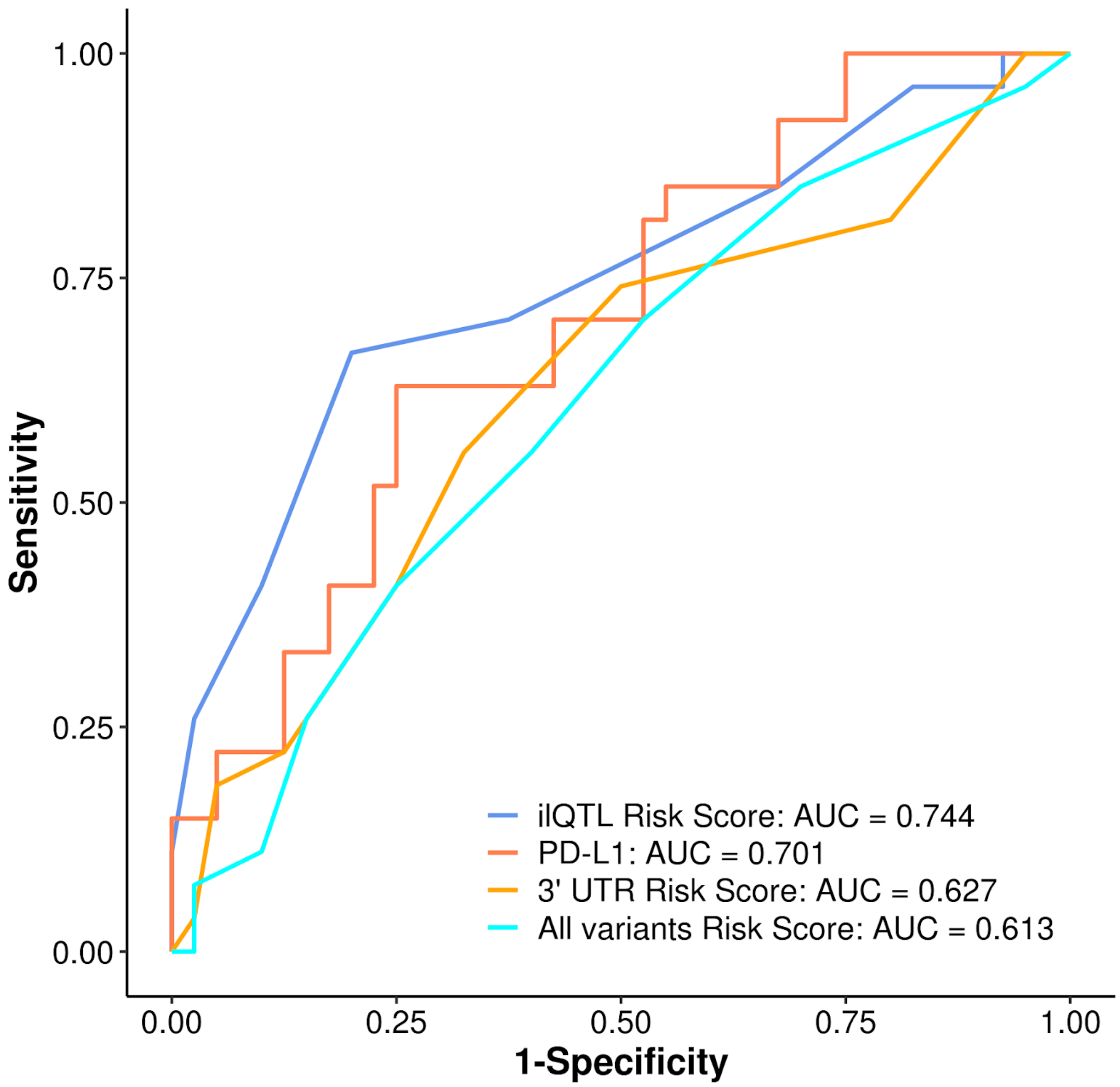

Supplementary Figure 9. Comparison of ilQTL 3' UTR PRS with 3' UTR and genome-wide PRS in distinguishing responders and non-responders to ICI. Receiver Operator Characteristic (ROC) curves showing the ability of the PRS score classifications, calculated based on significantly top enriched variants from (i) ilQTLs (n=28, blue), (ii) 3' UTR (n=28, orange), (iii) all enriched variants in CDS and UTR regions (n=28, cyan), coupled with PD-L1 expression classification to distinguish between R and NR patients in the testing population (n=67). An Area Under the Curve (AUC) score is reported for all models. Figure data are provided in the Source Data file.

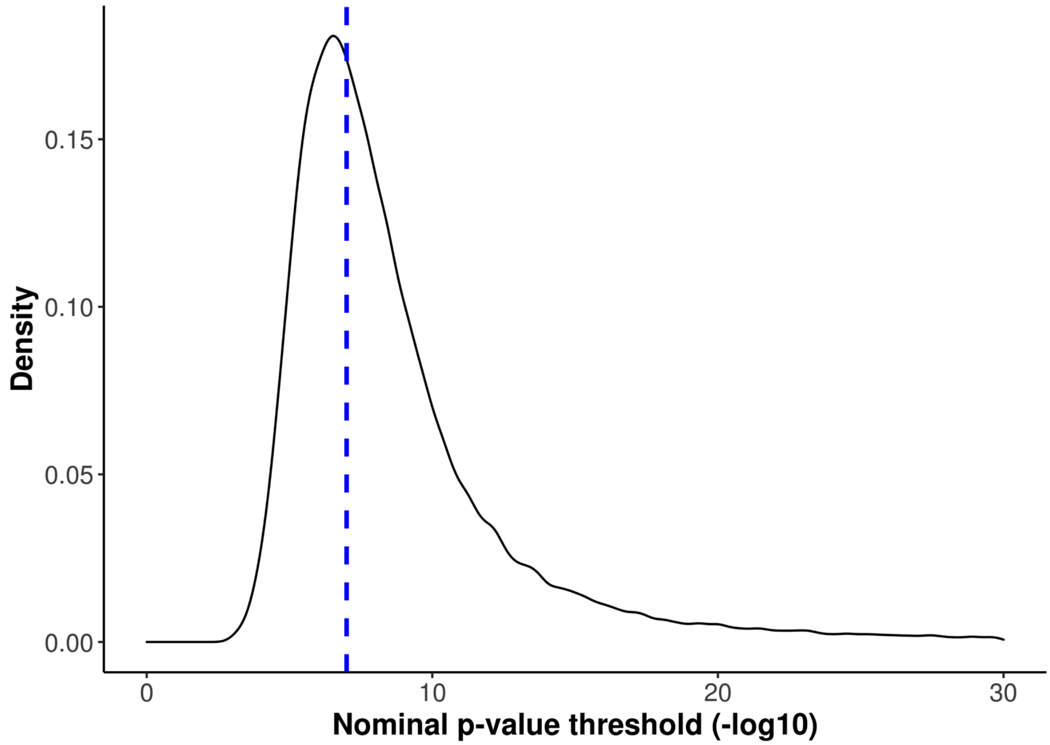

Supplementary Figure 10. Nominal p-value threshold for genes with at least one significant variant. Density plot showing the distribution of nominal p-value thresholds for genes with a significant q-value (<0.05). The blue line represents x=7 corresponding to a nominal p-value of 1e-7.

Supplementary Table 1. RBP binding sites overlapping with ADAR variant (chr1: 154583325, T-to-C)

chr	start	end	strand	Database	RBP	Type of evidence	Cell line	GSE study	Score
chr1	154583282	154583348	-	human_RBP_CLIPdb_2630524	ELAVL1P	AR-CLIP,PARalyzer	HEK293	GSE28865,GSM714639	0.966599073
chr1	154583289	154583345	-	human_RBP_CLIPdb_2630528	HNRNPCP	AR-CLIP,PARalyzer	HEK293T	GSE56010,GSM1350197	0.989801254
chr1	154583289	154583351	-	human_RBP_CLIPdb_2630529	HNRNPCP	AR-CLIP,PARalyzer	HEK293T	GSE56010,GSM1350199	0.988528479
chr1	154583292	154583372	-	human_RBP_CLIPdb_2630530	CPSF7P	AR-CLIP,PARalyzer	HEK293	GSE37401,GSM917663	0.992829603
chr1	154583293	154583351	-	human_RBP_CLIPdb_2630531	HNRNPCP	AR-CLIP,PARalyzer	HEK293T	GSE56010,GSM1350201	0.970522495
chr1	154583297	154583355	-	human_RBP_CLIPdb_2630534	MOV10P	AR-CLIP,PARalyzer	HEK293	GSE37524,GSM921128	0.747499594
chr1	154583299	154583351	-	human_RBP_CLIPdb_2630535	HNRNPCP	AR-CLIP,PARalyzer	HEK293T	GSE56010,GSM1350196	0.559343792
chr1	154583300	154583330	-	human_RBP_CLIPdb_2630536	TARDBP	AR-CLIP,PARalyzer	HEK293T	DRA001158,DRS012387	0.695848418
chr1	154583303	154583330	-	human_RBP_CLIPdb_2630537	ELAVL1P	AR-CLIP,PARalyzer	HEK293	GSE50989,GSM1234284	0.628681354
chr1	154583304	154583333	-	human_RBP_CLIPdb_2630547	HNRNPCP	AR-CLIP,PARalyzer	HEK293T	GSE56010,GSM1350200	0.612743743
chr1	154583304	154583344	-	human_RBP_CLIPdb_2630548	PTBP1	iCLIP,Piranha_0.01	HEK293T	GSE57278,GSM1378377	16
chr1	154583305	154583349	-	human_RBP_CLIPdb_2630549	HNRNPCP	AR-CLIP,PARalyzer	HEK293T	GSE56010,GSM1350198	0.800485096
chr1	154583307	154583401	-	human_RBP_CLIPdb_2630550	CPSF6P	AR-CLIP,PARalyzer	HEK293	GSE37401,GSM917665	0.835541102
chr1	154583310	154583358	-	human_RBP_CLIPdb_2630551	ZC3H7BP	AR-CLIP,PARalyzer	HEK293	GSE38201,GSM936510	0.709283524
chr1	154583323	154583345	-	human_RBP_CLIPdb_2630552	IGF2BP3	PAR-CLIP,PARalyzer	HEK293	GSE21578,GSM545209	0.62220225
chr1	154583323	154583351	-	human_RBP_CLIPdb_2630553	FMR1	PAR-CLIP,PARalyzer	HEK293	GSE39682,GSM977615	0.792110063
chr1	154583323	154583369	-	human_RBP_CLIPdb_2630554	ATXN2P	AR-CLIP,PARalyzer	HEK293T	DRA001158,DRS012391	0.751775938
chr1	154583324	154583344	-	human_RBP_CLIPdb_2630555	UPF1	HITS-CLIP,Piranha_0.01	HEK_293_FRT	GSE69586,GSM1704214	22
chr1	154583325	154583346	-	human_RBP_CLIPdb_2630556	TIAL1	iCLIP,CIMS	HeLa	E-MTAB-432,ERR039788-ERR039789	5

Supplementary Table 2. Predicted miRNA binding sites overlapping with ADAR variant (chr1: 154583325, T-to-C)

chr	position	variant_id	chr_binding_site	start_binding_site	end_binding_site	miRNA
chr1	154583325	chr1_154583325_T_C	chr1	154583305	154583325	hsa-miR-1289
chr1	154583325	chr1_154583325_T_C	chr1	154583305	154583330	hsa-miR-3198
chr1	154583325	chr1_154583325_T_C	chr1	154583297	154583325	hsa-miR-767-3p

Supplementary Table 3. TARDBP PAR-CLIP-defined binding site overlapping ADAR variant (chr1: 154583325, T-to-C)

**Start**	**End**	**Peak Sequence/Binding Motif**
chr1:154583300	chr1:154583330	GCTCTTGGAGTCATGACCAACACTCTAAAAG

Supplementary Table 4. List of variants included in the ilQTL PRS for immune-checkpoint response.

uniqueID	ilQTL	Gene name	Gene stable ID	Chr	start	end	Strand	Ref	Alt	ilQTL pval	Enrichment Qvalue
chr1_89181839_T_TA	Macrophages.M1	GBP4	ENSG00000162654	chr1	89181839	89181840	-	T	TA	1.11E-08	0.0055739
chr8_100920607_C_CA	Eosinophils	YWHAZ	ENSG00000164924	chr8	100920607	100920608	-	C	CA	7.83E-07	0.0025162
chr2_96185425_G_GA	T.cells.CD8	STARD7	ENSG00000084090	chr2	96185425	96185426	-	G	GA	1.07E-07	0.002614
chr13_36303938_A_G	T.cells.gamma.delta	SPART	ENSG00000133104	chr13	36303938	36303939	-	A	G	6.54E-06	0.0007032
chr13_77895645_T_TA	T.cells.gamma.delta	EDNRB	ENSG00000136160	chr13	77895645	77895646	-	T	TA	5.93E-15	4.89E-12
chr2_42350673_T_C	Eosinophils	COX7A2L	ENSG00000115944	chr2	42350673	42350674	-	T	C	5.07E-16	2.59E-11
chr13_42320277_G_T	TCR.Shannon	AKAP11	ENSG00000023516	chr13	42320277	42320278	+	G	T	3.52E-06	0.0463555
chr8_11842904_T_C	TCR.Shannon	CTSB	ENSG00000164733	chr8	11842904	11842905	-	T	C	1.70E-07	0.0054067
chr8_11843848_T_C	Macrophages.M1	CTSB	ENSG00000164733	chr8	11843848	11843849	-	T	C	1.06E-11	3.72E-05
chr1_155883332_A_G	T.cells.regulatory	SYT11	ENSG00000132718	chr1	155883332	155883333	+	A	G	4.69E-06	0.0462833
chr11_119663035_TT_AG	Eosinophils	NECTIN1	ENSG00000110400	chr11	119663035	119663036	-	TT	AG	1.56E-05	0.024478
chr2_42350666_T_A	TCR.Shannon	COX7A2L	ENSG00000115944	chr2	42350666	42350667	-	T	A	1.21E-07	0.0041344
chrX_23702029_T_C	T.cells.gamma.delta	ACOT9	ENSG00000123130	chrX	23702029	23702030	-	T	C	7.97E-12	3.91E-09
chr3_44749367_T_C	TCR.Shannon	KIAA1143	ENSG00000163807	chr3	44749367	44749368	-	T	C	8.62E-09	0.0005432
chr21_33357771_A_G	Macrophages.M1	IFNAR1	ENSG00000142166	chr21	33357771	33357772	+	A	G	1.11E-07	0.0352439
chr5_77691221_G_T	NK.cells.resting	TBCA	ENSG00000171530	chr5	77691221	77691222	-	G	T	6.62E-08	0.017185
chr2_203300670_A_G	T.cells.regulatory	CYP20A1	ENSG00000119004	chr2	203300670	203300671	+	A	G	3.72E-08	0.0013719
chr17_44035695_G_A	Eosinophils	LSM12	ENSG00000161654	chr17	44035695	44035696	-	G	A	1.58E-05	0.0247163
chr17_5441165_T_A	Eosinophils	DHX33	ENSG00000005100	chr17	5441165	5441166	-	T	A	1.24E-07	0.0005835
chr6_8014553_T_C	T.cells.gamma.delta	BLOC1S5	ENSG00000188428	chr6	8014553	8014554	-	T	C	3.33E-06	0.0003964
chr1_167420226_T_C	Eosinophils	POU2F1	ENSG00000143190	chr1	167420226	167420227	+	T	C	4.14E-05	0.048019
chr4_88260326_T_C	T.cells.gamma.delta	PPM1K	ENSG00000163644	chr4	88260326	88260327	-	T	C	9.85E-14	6.67E-11
chr22_18088653_A_G	Eosinophils	PEX26	ENSG00000215193	chr22	18088653	18088654	+	A	G	1.94E-07	0.0008403
chr2_127845576_T_C	Eosinophils	POLR2D	ENSG00000144231	chr2	127845576	127845577	-	T	C	1.01E-07	0.000497
chr14_96089980_C_T	T.cells.gamma.delta	C14orf132	ENSG00000227051	chr14	96089980	96089981	+	C	T	2.30E-10	8.52E-08
chr17_68421262_T_A	T.cells.gamma.delta	ARSG	ENSG00000141337	chr17	68421262	68421263	+	T	A	1.99E-09	6.00E-07
chr5_157740793_A_G	T.cells.gamma.delta	THG1L	ENSG00000113272	chr5	157740793	157740794	+	A	G	0.000817	0.0410857
chr14_92071043_TT_GC	Eosinophils	ATXN3	ENSG00000066427	chr14	92071043	92071044	-	TT	GC	8.12E-06	0.0154123
