# Supplementary material for: Determinants of gastric cancer immune escape identified from non-coding immune-landscape quantitative trait loci
Source: Nat Commun. 2024 May 21;15:4319. doi: 10.1038/s41467-024-48436-5 (PMC11109163; doi:10.1038/s41467-024-48436-5)
Supplement: Supplementary file 3 — Description of Additional Supplementary Files [file 41467_2024_48436_MOESM3_ESM.pdf]

Description of Additional Supplementary Files

File Name: Supplementary Data 1
Description: TCGA IDs, sex and self-reported race information about STAD patients included in the analysis

File Name: Supplementary Data 2
Description: List of immune-related genes.

File Name: Supplementary Data 3
Description: Immune-related 3'UTR cis-eQTLs (pval_nominal < 10^-5)

File Name: Supplementary Data 4
Description: MPRA assay results.

File Name: Supplementary Data 5
Description: List of primers used in MPRA assay.
