# Supplementary material for: Determinants of gastric cancer immune escape identified from non-coding immune-landscape quantitative trait loci
Source: Nat Commun. 2024 May 21;15:4319. doi: 10.1038/s41467-024-48436-5 (PMC11109163; doi:10.1038/s41467-024-48436-5)
Supplement: Supplementary file 5 — Reporting Summary [file 41467_2024_48436_MOESM5_ESM.pdf]

nature portfolio

Corresponding author(s): Frank J. Slack and Ioannis S. Vlachos

Last updated by author(s): Apr 29, 2024

Reporting Summary

Nature Portfolio wishes to improve the reproducibility of the work that we publish. This form provides structure for consistency and transparency in reporting. For further information on Nature Portfolio policies, see our Editorial Policies and the Editorial Policy Checklist.

Statistics

For all statistical analyses, confirm that the following items are present in the figure legend, table legend, main text, or Methods section.

n/a	Confirmed	
☐	☒	The exact sample size (n) for each experimental group/condition, given as a discrete number and unit of measurement
☐	☒	A statement on whether measurements were taken from distinct samples or whether the same sample was measured repeatedly
☐	☒	The statistical test(s) used AND whether they are one- or two-sided<br>*Only common tests should be described solely by name; describe more complex techniques in the Methods section.*
☐	☒	A description of all covariates tested
☐	☒	A description of any assumptions or corrections, such as tests of normality and adjustment for multiple comparisons
☐	☒	A full description of the statistical parameters including central tendency (e.g. means) or other basic estimates (e.g. regression coefficient) AND variation (e.g. standard deviation) or associated estimates of uncertainty (e.g. confidence intervals)
☐	☒	For null hypothesis testing, the test statistic (e.g. F, t, r) with confidence intervals, effect sizes, degrees of freedom and P value noted<br>*Give P values as exact values whenever suitable.*
☒	☐	For Bayesian analysis, information on the choice of priors and Markov chain Monte Carlo settings
☐	☒	For hierarchical and complex designs, identification of the appropriate level for tests and full reporting of outcomes
☐	☒	Estimates of effect sizes (e.g. Cohen's d, Pearson's r), indicating how they were calculated

Our web collection on statistics for biologists contains articles on many of the points above.

Software and code

Policy information about availability of computer code

Data collection	*Provide a description of all commercial, open source and custom code used to collect the data in this study, specifying the version used OR state that no software was used.*
Data analysis	Computational analyses are described in detail in the methods section, including versions for software. The majority of analyses have been performed utilizing publicly available tools. Custom code utilized for the MPRA analysis has been deposited at https://github.com/ivlachos/3UTR.

For manuscripts utilizing custom algorithms or software that are central to the research but not yet described in published literature, software must be made available to editors and reviewers. We strongly encourage code deposition in a community repository (e.g. GitHub). See the Nature Portfolio guidelines for submitting code & software for further information.

Data

Policy information about availability of data

All manuscripts must include a data availability statement. This statement should provide the following information, where applicable:

- Accession codes, unique identifiers, or web links for publicly available datasets
- A description of any restrictions on data availability
- For clinical datasets or third party data, please ensure that the statement adheres to our policy

The data that support the findings of this study are derived from publicly available datasets. Raw RNAseq, WES, and SNP array data for gastric adenocarcinoma patients were obtained from The Cancer Genome Atlas (TCGA) database at gdc.cancer.gov. The TCGA barcodes of the STAD patients included in the study are

provided in the Supplementary Information. The PRS and predictive model analyses were performed on a combined cohort of publically available melanoma and gastric cancer pre-treatment RNAseq data obtained from the Gene expression Omnibus (GEO) database under accession codes GSE11582150 (https://www.ncbi.nlm.nih.gov/geo/query/acc.cgi?acc=GSE115821), GSE7822051 (https://www.ncbi.nlm.nih.gov/geo/query/acc.cgi?acc=gse78220), and GSE9106169 (https://www.ncbi.nlm.nih.gov/geo/query/acc.cgi?acc=GSE91061), and the European Nucleotide Archive (ENA) under accession PRJEB2578070 (https://www.ncbi.nlm.nih.gov/bioproject/?term=PRJEB25780). Matched WES data for patients included in the PRS and predictive model analyses were obtained from the Sequence Read Archive (SRA, https://www.ncbi.nlm.nih. gov/sra) under accessions SRP067938 (https://www.ncbi.nlm.nih.gov/sra/?term=SRP067938) and SRP09029451 (https://www.ncbi.nlm.nih.gov/sra/?term=SRP090294) and from ENA under accession ERP10773470 (http://www.ebi.ac.uk/ena/data/view/ERP107734). The raw MPRA amplicon sequencing data generated in this study have been deposited in the GEO database under accession code GSE261709 (https://www.ncbi.nlm.nih.gov/geo/query/acc.cgi?acc=GSE261709). The remaining data are available within the Article, Supplementary Information or Source Data file.

Research involving human participants, their data, or biological material

Policy information about studies with human participants or human data. See also policy information about sex, gender (identity/presentation), and sexual orientation and race, ethnicity and racism.

Reporting on sex and gender: Self-reported sex was used as a covariate in our analysis.

Reporting on race, ethnicity, or other socially relevant groupings: Genetic principal components that correlated with self-reported race were used as covariates in our analysis.

Population characteristics: Racially diverse cancer patient population

Recruitment: N/A

Ethics oversight: N/A

Note that full information on the approval of the study protocol must also be provided in the manuscript.

Field-specific reporting

Please select the one below that is the best fit for your research. If you are not sure, read the appropriate sections before making your selection.

[x] Life sciences [ ] Behavioural & social sciences [ ] Ecological, evolutionary & environmental sciences

For a reference copy of the document with all sections, see nature.com/documents/nr-reporting-summary-flat.pdf

Life sciences study design

All studies must disclose on these points even when the disclosure is negative.

Sample size: Raw RNAseq data were obtained for 375 TCGA STAD primary cancer and 40 matched control normal tissue samples. It is the largest available cohort of gastric cancer patients with concordant RNAseq and clinical data f, making it the best source for this study. No sample-size calculation was performed. Prior eQTL study experience and publications such as GTEx pointed that 375 patients could provide adequate power for the detection of eQTL events.

Data exclusions: No data were excluded.

Replication: Experiments were repeated in triplicate and and all replicates are reported in the results.

Randomization: Patients from the immune checkpoint inhibitor studies were randomly allocated into training/testing cohorts by following best practices.

Blinding: Blinding is not applicable to this study design (eQTL/QTL analysis).

Reporting for specific materials, systems and methods

We require information from authors about some types of materials, experimental systems and methods used in many studies. Here, indicate whether each material, system or method listed is relevant to your study. If you are not sure if a list item applies to your research, read the appropriate section before selecting a response.

Materials & experimental systems

n/a	Involved in the study
x	Antibodies
	x Eukaryotic cell lines
x	Palaeontology and archaeology
x	Animals and other organisms
x	Clinical data
x	Dual use research of concern
x	Plants

Methods

n/a	Involved in the study
x	ChIP-seq
x	Flow cytometry
x	MRI-based neuroimaging

Eukaryotic cell lines

Policy information about cell lines and Sex and Gender in Research

Cell line source(s)	AGS (ATCC) and SNU719 (KCLB)
Authentication	The cell lines were verified by the vendors by STR profiling.
Mycoplasma contamination	Cell lines tested negative for mycoplasma contamination at regular intervals.
Commonly misidentified lines (See ICLAC register)	The cell lines are not part of the ICLAC list of commonly misidentified lines.

Plants

Seed stocks	*Report on the source of all seed stocks or other plant material used. If applicable, state the seed stock centre and catalogue number. If plant specimens were collected from the field, describe the collection location, date and sampling procedures.*
Novel plant genotypes	*Describe the methods by which all novel plant genotypes were produced. This includes those generated by transgenic approaches, gene editing, chemical/radiation-based mutagenesis and hybridization. For transgenic lines, describe the transformation method, the number of independent lines analyzed and the generation upon which experiments were performed. For gene-edited lines, describe the editor used, the endogenous sequence targeted for editing, the targeting guide RNA sequence (if applicable) and how the editor was applied.*
Authentication	*Describe any authentication procedures for each seed stock used or novel genotype generated. Describe any experiments used to assess the effect of a mutation and, where applicable, how potential secondary effects (e.g. second site T-DNA insertions, mosiacism, off-target gene editing) were examined.*
